# The Cardiorespiratory Demands of Treadmill Walking with and without the Use of Ekso GT™ within Able-Bodied Participants: A Feasibility Study

**DOI:** 10.3390/ijerph19106176

**Published:** 2022-05-19

**Authors:** Damien Duddy, Rónán Doherty, James Connolly, Johnny Loughrey, Joan Condell, David Hassan, Maria Faulkner

**Affiliations:** 1 Sports Lab North West, Atlantic Technological University Donegal, Letterkenny Campus, Port Road, F92 FC93 Letterkenny, Ireland; ronan.doherty@atu.ie (R.D.); maria.faulkner@atu.ie (M.F.); 2 Department of Computing, Atlantic Technological University Donegal, Letterkenny Campus, Port Road, F92 FC93 Letterkenny, Ireland; james.connolly@atu.ie; 3 The No Barriers Foundation, F92 TW27 Letterkenny, Ireland; johnny@jtphysio.com; 4 School of Computing, Engineering and Intelligent Systems, Ulster University Magee, Londonderry BT48 7JL, UK; j.condell@ulster.ac.uk; 5 Sport and Exercise Sciences Research Institute, Ulster University Jordanstown, Newtownabbey BT37 0QB, UK; d.hassan@ulster.ac.uk

**Keywords:** exoskeleton, cardiorespiratory fitness, neurological impairments, physical activity, oxygen consumption, quality of life

## Abstract

Individuals with neurological impairments tend to lead a predominantly sedentary lifestyle due to impaired gait function and mobility. This may be detrimental to health by negatively impacting cardiorespiratory fitness and muscular strength, and increasing the risk of developing secondary health problems. Powered exoskeletons are assistive devices that may aid neurologically impaired individuals in achieving the World Health Organisation’s (WHO) physical activity (PA) guidelines for health. Increased PA should elicit a sufficient cardiorespiratory stimulus to provide health benefits to exoskeleton users. This study examined the cardiorespiratory demands of treadmill walking with and without the Ekso GT™ among able-bodied participants. The Ekso GT™ is a powered exoskeleton that enables individuals with neurological impairments to walk by supporting full body mass with motors attached at the hip and knee joints to generate steps. This feasibility study consisted of one group of healthy able-bodied individuals (*n* = 8). Participants completed two 12 min treadmill walking assessments, one with and one without the Ekso GT™ at the same fixed speed. Throughout each walking bout, various cardiorespiratory parameters, namely, volume of oxygen per kilogram (kg) of body mass ( $\dot{V}$ O_2_·kg^−1^), volume of carbon dioxide per kg of body mass ( $\dot{V}$ CO_2_·kg^−1^), respiratory exchange ratio (RER), ventilation ( $\dot{V}$ _E_), heart rate (HR), and rate of perceived exertion (RPE), were recorded. Treadmill walking with Ekso GT™ elevated all recorded measurements to a significantly greater level (*p* ≤ 0.05) (except RER at 1 km·h^–1^; *p =* 0.230) than treadmill walking without the Ekso GT™ did at the same fixed speed. An increased cardiorespiratory response was recorded during treadmill walking with the exoskeleton. Exoskeleton walking may, therefore, be an effective method to increase PA levels and provide sufficient stimulus in accordance with the PA guidelines to promote cardiorespiratory fitness and subsequently enhance overall health.

## 1. Introduction

Globally, spinal cord injury (SCI) affects approximately 0.93 million individuals [1], 13.7 million strokes occur annually [2], and associated disabilities often result in reduced gait function [3,4]. This may negatively impact independence, social roles [5], and quality of life (QoL) [6]. Individuals with neurological impairments often rely on a wheelchair as their primary source of mobility. Consequently, wheelchair use alone may not result in an adequate amount of physical activity (PA) per day, leading to a sedentary lifestyle [7,8], which is defined as any waking behaviour that equates to ≤1.5 metabolic equivalents while in a supine, reclined, or seated position [9]. Therefore, neurologically compromised individuals may be more susceptible to physical deconditioning, resulting in reduced cardiorespiratory fitness and muscular strength [7]. Additionally, this population is at an increased risk of developing secondary health problems, such as muscular spasticity, pressure ulcers and osteoporosis, as well as psychosomatic, metabolic, and cardiorespiratory diseases, subsequently increasing the risk of mortality [10,11].

According to the World Health Organisation’s (WHO) PA guidelines for health, adults with disabilities should aim to complete at least 150–300 min of moderate-intensity PA or 75–150 min of vigorous-intensity PA, or an equivalent combination of both per week [12]. Adhering to the WHO, PA guidelines may improve cardiorespiratory fitness, physical and mental functioning, upper-extremity function, and health-related QoL, reduce the risk of physical deconditioning, and diminish previously discussed secondary health conditions [12,13]. The most common method used to achieve moderate-intensity activity among neurologically impaired individuals is an arm crank or wheelchair ergometer. Therefore, muscle mass used to perform these exercises is primarily located in the upper extremities [14]. However, powered exoskeletons have recently emerged as a new rehabilitation method. Exoskeletons enable individuals with limited gait function to walk overground by providing maximal external support, and replicating the lower-extremity movement and weight-bearing patterns of an able-bodied individual [14,15].

Previous research suggested that powered exoskeletons may provide adequate stimulus to enhance cardiorespiratory function [16], mainly by elevating oxygen consumption and heart rate (HR) [17,18]. However, these studies investigated overground walking with an Indego exoskeleton [16] and a hybrid neuroprosthesis exoskeleton [17]. Powered exoskeletons also improve walking ability [19,20], balance, strength, breathing, circulation, bowel, and urinary tract function, and reduce pain and spasticity [13]. Improvements in body perception, sleep quality, mood, and several social benefits were reported by exoskeleton users [13,21]. Depending on injury severity, the PA guidelines may not be achievable; therefore, adults with disabilities should start by completing small amounts of PA and gradually increase the frequency, intensity, and duration [12]. Exoskeleton rehabilitation may enable the user to achieve moderate-intensity PA and adhere to the PA guidelines more effectively than wheelchair propulsion or an arm crank can. The purpose of this study was to examine the cardiorespiratory demands of treadmill walking with and without the exoskeleton at various walking speeds within an able-bodied population, and to ensure that the chosen methodology was safe to be applied to neurologically impaired populations.

## 2. Materials and Methods

### 2.1. Participants

Participants were recruited from Atlantic Technological University (ATU) Donegal, Letterkenny Campus, and “The No Barriers Foundation” in accordance with inclusion criteria in a crossover study design (Table 1). Prior to participation, all individuals received a participant information sheet containing information about the project, and completed a health history questionnaire and consent form. Additionally, prior to taking part, all participants received a comprehensive explanation and demonstration about how the exoskeleton works, and a question-and-answer session was conducted for further clarification. All procedures were approved by the ATU Donegal, Letterkenny Campus research ethics committee prior to the collection of any data. In line with current general data protection regulation [22], all data were coded, and participant names were replaced with identification codes to ensure anonymity [23]. All testing was conducted in Sports Lab North West within ATU Donegal, Letterkenny Campus. Standard operating procedures were followed for each test, and at least two assistants were present during the testing sessions.

### 2.2. Anthropometric Measurements

Height (cm) was recorded using a stadiometer (Seca 213 stadiometer, GMBH, Hamburg, Germany), and body mass (kg) was recorded using Seca weighing scales (Seca GMBH & Co. kg., Hamburg, Germany, Model: 899 7021094).

### 2.3. Outcome Measures

#### 2.3.1. Ekso GT™

**Figure 1:**
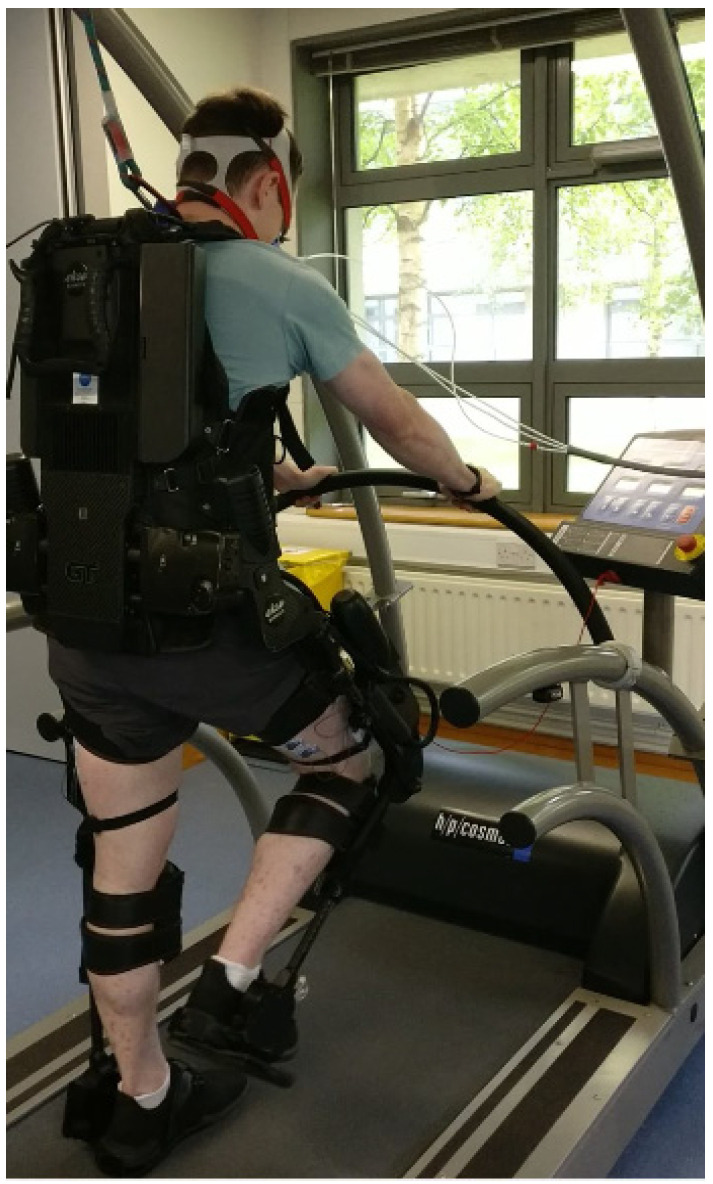
Ekso GT™ during treadmill walking.

Participants were secured into the Ekso GT™ (Richmond, CA, USA) with a backpack-style shoulder harness and a torso brace (Figure 1). Participant’s legs were fixed in place with thigh straps, shin guards, and secure foot binding. To fit the participants into the device, the Ekso GT™ was adjusted at the hips to align with the distance between the two greater trochanters, and femur length was adjusted on the device to accommodate the distance between the greater trochanters and the lateral joint line of the knee. The lower leg segment of the Ekso GT™ was adjusted to align with the distance between the lateral joint of the knee and the bottom of the foot. Ankle stiffness of the suit was also adjusted when required depending on the individuals’ mobility.

#### 2.3.2. Cardiorespiratory Measurements

The Ganshorn PowerCube^®^ (Ganshorn, Niederlauer, Germany) was employed to record cardiorespiratory measurements. The Ganshorn PowerCube^®^ generated cardiorespiratory variables, i.e., volume of oxygen consumption per kilogram (kg) of body mass ( $\dot{V}$ O_2_·kg^−1^; mL·kg^−1^·min^−1^), volume of carbon dioxide per kg of body mass ( $\dot{V}$ CO_2_·kg^−1^; mL·kg^−1^·min^−1^), respiratory exchange ratio (RER), and ventilation ( $\dot{V}$ _E_; L) by conducting breath-by-breath analysis of exchanged respiratory gases. Prior to testing, the Ganshorn PowerCube^®^ was calibrated in accordance with the manufacturer instructions. HR (bpm) data were collected using a polar HR monitor (Polar FT2 GEN 90037558, Electro, Finland) attached to the participants chest, and HR data signals were transmitted to the treadmill receiver. Cardiorespiratory data generated by the Ganshorn PowerCube^®^ were collected once per minute, and HR data were recorded every 30 s during both walking assessments with and without the Ekso GT™.

#### 2.3.3. Rate of Perceived Exertion

RPE was assessed using a Borg scale in the range of 6–20, with 6 labelled as “no exertion at all”, and 20 equating to “maximal exertion” [24,25]. RPE was collected during the last 15 s of each minute during both treadmill walking bouts.

### 2.4. Treadmill Walking Protocols

#### 2.4.1. Rest and Recovery

Participants completed two 12 min bouts of treadmill walking: one without the Ekso GT™ and one with the Ekso GT™. Resting HR (RHR; bpm) and resting blood pressure (RBP; mmHg) were recorded prior to both walking periods. Participants were given a five-minute rest period prior to the first walking bout without the Ekso GT™ to ensure that resting values were achieved. Previous research suggested a one-to-one work-to-rest ratio for aerobic activity [26,27]; therefore, a 12 min rest period was provided prior to the second walking bout, i.e., using the Ekso GT™. Throughout the first five-minute rest period, RHR was recorded every 30 s, and RBP was recorded a total of three times immediately after the rest period using a Nissei digital blood pressure (BP) monitor (DS-1902). During the last five minutes of the second rest period prior to walking with the exoskeleton, RHR (bpm) was collected every 30 s, and RBP (mmHg) was recorded a further three times immediately after the rest period to ensure that HR and BP had returned to estimated resting values.

#### 2.4.2. Treadmill Walking

Both walking bouts, i.e., with and without the exoskeleton, were completed at the same fixed speed during the 12 min progressive treadmill assessment, which consisted of three consecutive four-minute phases at 1, 2, and 3 km·h^−^^1^. Four minutes of walking were selected at each speed to ensure participants reached a steady-state plateau in response to an increase in exercise intensity [28]. As the participants were able-bodied, the Ekso GT™ was in the most active mode throughout to enable participants to move freely without assistance from the exoskeleton. To prevent accidental falls, participants wore an overhead harness during both walking bouts. It was also necessary for participants to hold onto the front rail of the treadmill when using the exoskeleton for health and safety reasons. Therefore, participants also held onto the front rail when walking without the exoskeleton to accurately replicate and standardise walking protocols. Submaximal tests were selected to ensure they were safe and replicable within a neurologically compromised cohort.

### 2.5. Statistical Analysis

All data were statistically analysed using SPSS software version 26 (SPSS Inc., Chicago, IL, USA). Descriptive data are displayed as mean ± standard deviation (SD). To ensure that the data were representative of the steady-state plateau following increased exercise intensity, mean data from the last three minutes of walking at each speed, both with and without the Ekso GT™, were selected for analysis. Any missing data were replaced with the series mean for each minute. The Shapiro–Wilk test was employed to test data normality as it is the most accurate method to detect non-normality within a small sample (*n* < 50) [29]. Q–Q plots were used to visually verify the distribution outcome determined by the Shapiro–Wilk test. Normally distributed data were analysed using the paired-samples *t*-test. The Wilcoxon signed-rank test was used to analyse nonparametrically distributed data to compare cardiorespiratory demands of treadmill walking with and without the Ekso GT™. Confidence intervals were set at 95%, and a *p* value of ≤0.05 was deemed to be statistically significant. G*Power software (version 3.1.9.7, Franz Faul, Kiel, Germany) was employed to conduct two-tailed power analysis and calculate the required sample size. The employed statistical test was means difference between two dependent means (matched pairs). Effect size (ES) was determined using $\dot{V}$ O_2_ means and SDs collected by Asselin et al. [30] (11.2 ± 1.7 mL·kg^−1^·min^−1^), and Maher et al. [18] (8.5 ± 0.90 mL·kg^−1^·min^−1^) during powered exoskeleton assisted walking. The ES was 1.83, and power was set at 0.95. A total of seven participants were required with an actual study power of 0.98. Cohen’s *d* formula: (M_2_ – M_1_)/SD_pooled_, was used to calculate the ES of the current findings [31]. To determine the strength of the ES, results were compared to a scoring system where 0.2 = small, 0.5 = medium, and 0.8 = large effect [32].

## 3. Results

One group of healthy able-bodied individuals (*n* = 8) participated in this crossover study design. Participants comprised seven males (*n* = 7) and one female (*n* = 1). Participant information such as age (25.5 ± 5.2 years; range: 20–33 years), height (178.5 ± 5.9 cm; range: 170–186.5 cm), and body mass (82.9 ± 9.7 kg; range: 66.4–96.1 kg) were collected. According to the Shapiro–Wilk test and Q–Q plots, all data were normally distributed (*p* > 0.05) except RER when walking without the Ekso GT™ at 2 km·h^−1^, RPE when walking without the Ekso GT™ at all speeds and RPE when walking with the Ekso GT™ at 3 km·h^−1^.

### 3.1. RBP and RHR

Prior to walking without the Ekso GT™, mean RBP was 123 ± 7/77 ± 9 mmHg, which reduced to 122 ± 13/77 ± 11 mmHg prior to walking with the Ekso GT™. Mean RHR was 70 ± 20 bpm prior to non-exoskeleton treadmill walking, and 69 ± 20 bpm when repeated before walking with the exoskeleton. 

### 3.2. $\dot{V}$ O_2_·kg^−^^1^

**Figure 2:**
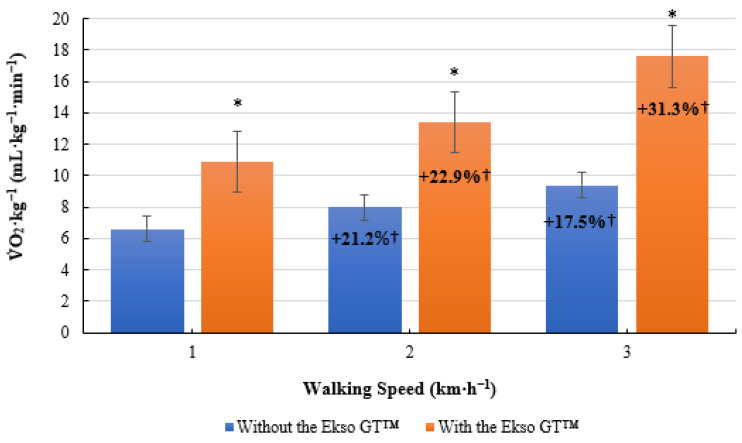
Mean volume of oxygen consumption per kilogram of body mass $(\dot{V}$ O_2_·kg^−^^1^; mL·kg^−1^·min^−1^) results during treadmill walking both with and without the Ekso GT™. * Significantly greater (*p* ≤ 0.05) when walking with Ekso GT™ versus walking without Ekso GT™. † Mean percentage $\dot{V}$ O_2_·kg^−1^ increase at each walking phase, both with and without Ekso GT™.

$\dot{V}$ O_2_·kg^−1^ was significantly higher when walking with the Ekso GT™ (10.9 ± 1.4 mL·kg^−1^·min^−1^, *p* ≤ 0.001; ES = 0.9, large) versus walking without the Ekso GT™ (6.6 ± 1.3 mL·kg^−1^·min^−1^) at 1 km·h^−1^. When walking at 2 km·h^−1^, $\dot{V}$ O_2_·kg^−1^ was significantly greater when using the Ekso GT™ (13.4 ± 1.4 mL·kg^−1^·min^−1^, *p* ≤ 0.001; ES = 0.9, large) in comparison to walking without the Ekso GT™ (8.0 ± 1.5 mL·kg^−1^·min^−1^). There was a significant difference in $\dot{V}$ O_2_·kg^−1^ results when comparing exoskeleton walking (17.6 ± 1.8 mL·kg^−1^·min^−^^1^, *p* ≤ 0.001; ES = 0.9, large) to non-exoskeleton walking (9.4 ± 1.4 mL·kg^−1^·min^−^^1^) at 3 km·h^−1^. Mean $\dot{V}$ O_2_·kg^−1^ percentage increase from 1 to 2 km·h^−1^ was greater when walking with the Ekso GT™ (22.9%) than that when walking without the Ekso GT™ (21.2%). A greater mean $\dot{V}$ O_2_·kg^−1^ percentage increase was also observed between 2 km·h^−1^ and 3 km·h^−1^ during exoskeleton (31.3%) versus non-exoskeleton (17.5%) treadmill walking (Figure 2).

### 3.3. $\dot{V}$ CO_2_·kg^−^^1^

**Figure 3:**
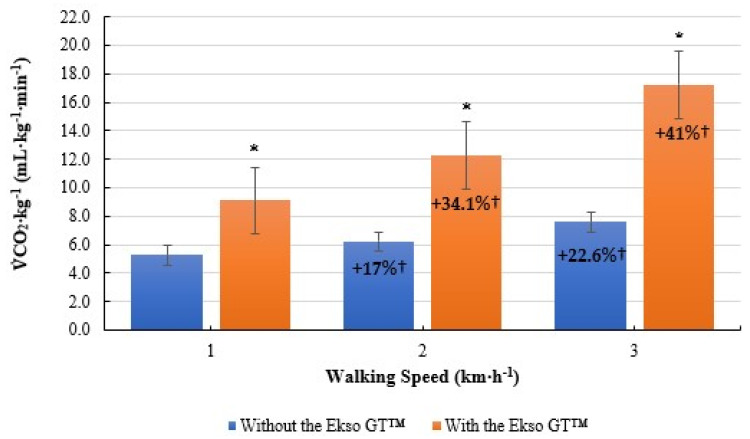
Mean volume of carbon dioxide per kilogram of body mass $(\dot{V}$ CO_2_·kg^−^^1^; mL·kg^−^^1^·min^−1^) results during treadmill walking both with and without Ekso GT™. * Significantly greater (*p* ≤ 0.05) when walking with Ekso GT™ versus walking without Ekso GT™. † Mean percentage $\dot{V}$ CO_2_·kg^−1^ increase at each walking phase, both with and without Ekso GT™.

Similarly, $\dot{V}$ CO_2_·kg^−1^ was significantly greater when walking with the Ekso GT™ (9.1 ± 1.5 mL·kg^−1^·min^−1^, *p* ≤ 0.001; ES = 0.9, large) versus without the Ekso GT™ (5.3 ± 0.7 mL·kg^−1^·min^−1^) at 1 km·h^−1^. When walking at 2 km·h^−1^, $\dot{V}$ CO_2_·kg^−1^ results were significantly higher when walking with Ekso GT™ (12.2 ± 2.0 mL·kg^−1^·min^−1^, *p* ≤ 0.001; ES = 0.9, large) than those when walking without the Ekso GT™ (6.2 ± 0.6 mL·kg^−1^·min^−1^). Furthermore, exoskeleton treadmill walking produced significantly greater $\dot{V}$ CO_2_·kg^−1^ results (17.2 ± 3.5 mL·kg^−1^·min^−1^, *p* ≤ 0.001; ES = 0.9, large) in comparison to non-exoskeleton treadmill walking (7.6 ± 0.9 mL·kg^−1^·min^−^^1^) at 3 km·h^−1^. Mean $\dot{V}$ CO_2_·kg^−1^ percentage increase from 1 km·h^−1^ to 2 km·h^−1^ was greater when using the exoskeleton (34.1%) versus walking without the exoskeleton (17%). Mean $\dot{V}$ CO_2_·kg^−1^ percentage increase from 2 km·h^−1^ to 3 km·h^−1^ was also higher when walking with the Ekso GT™ (41%) in comparison to without the Ekso GT™ (22.6%) (Figure 3).

### 3.4. RER

**Figure 4:**
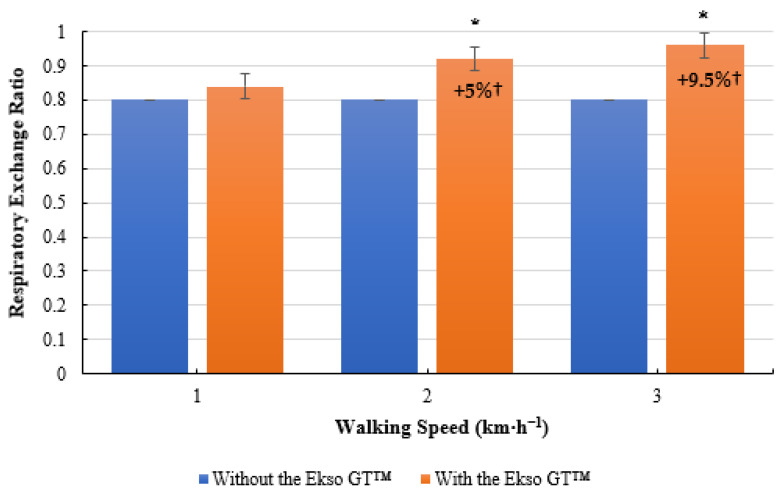
Mean respiratory exchange ratio (RER) results during treadmill walking with and without Ekso GT™. * Significantly greater (*p* ≤ 0.05) when walking with Ekso GT™ versus walking without Ekso GT™. † Mean percentage RER increase at each walking phase both with and without Ekso GT™.

No significant difference was present in RER scores when comparing treadmill walking with the Ekso GT™ (0.84 ± 0.13, *p* = 0.230; ES = 0.2, small) and treadmill walking without the Ekso GT™ (0.80 ± 0.10) at 1 km·h^−1^. RER was significantly greater when walking with the Ekso GT™ (0.92 ± 0.16, *p =* 0.012; ES = 0.4, small) versus walking without the Ekso GT™ (0.80 ± 0.11) at 2 km·h^−1^. Similarly, exoskeleton treadmill walking produced significantly higher RER results (0.96 ± 0.15, *p =* 0.006; ES = 0.6, medium) in comparison to non-exoskeleton treadmill (0.80 ± 0.07) walking at 3 km·h^−1^. Treadmill walking with the Ekso GT™ resulted in a mean percentage increase of 5% from 1 km·h^−1^ to 2 km·h^−1^, and a further increase of 9.5% between 2 and 3 km·h^−1^; no increase was recorded when walking without the Ekso GT™ (Figure 4).

### 3.5. $\dot{V}$ _E_

**Figure 5:**
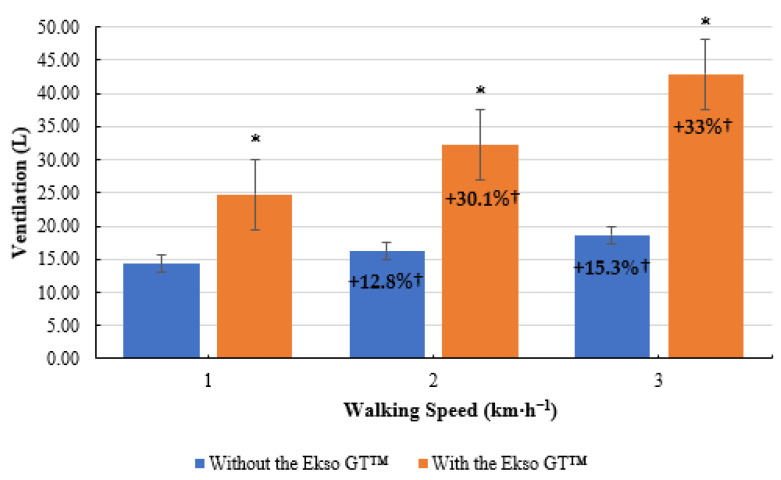
Mean ventilation ( $\dot{V}$ _E_) results during treadmill walking both with and without Ekso GT™. * Significantly greater (*p* ≤ 0.05) when walking with Ekso GT™ versus walking without Ekso GT™. † Mean percentage $\dot{V}$ _E_ increase at each walking phase with and without Ekso GT™.

Treadmill walking with the Ekso GT™ produced significantly higher $\dot{V}$ _E_ results (24.78 ± 3.66 L, *p* ≤ 0.001; ES = 0.9, large) than treadmill walking did without the Ekso GT™ (14.35 ± 1.40 L) at 1 km·h^−1^. When walking at 2 km·h^−1^, $\dot{V}$ _E_ was significantly greater during exoskeleton treadmill walking (32.24 ± 5.35 L, *p* ≤ 0.001; ES = 0.9, large) in comparison to non-exoskeleton treadmill walking (16.18 ± 1.66 L). Walking with the Ekso GT™ produced significantly greater $\dot{V}$ _E_ results (42.87 ± 8.07 L, *p* ≤ 0.001; ES = 0.9, large) compared to walking without the Ekso GT™ (18.65 ± 2.86 L) at 3 km·h^−1^. Mean $\dot{V}$ _E_ percentage increase between 1 km·h^−1^ and 2 km·h^−^^1^ was greater when walking with the Ekso GT™ (30.1%) versus walking without the Ekso GT™ (12.8%). A higher mean $\dot{V}$ _E_ percentage increase was also noted from 2 km·h^−1^ to 3 km·h^−1^ during exoskeleton walking (33%) compared to non-exoskeleton walking (15.3%) (Figure 5).

### 3.6. HR

**Figure 6:**
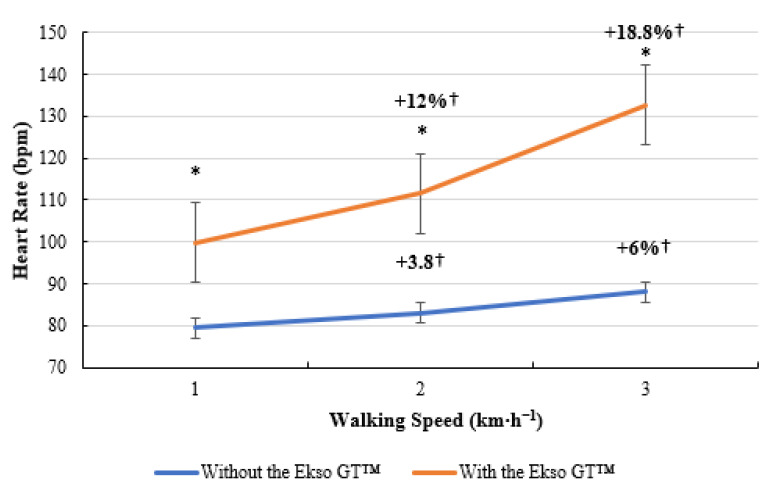
Heart rate (HR; bpm) data during treadmill walking with and without Ekso GT™. * Significantly greater (*p* ≤ 0.05) when walking with Ekso GT™ versus walking with Ekso GT™. † Mean percentage HR increase at each walking phase with and without Ekso GT™.

As illustrated below, a similar trend was present for HR data (Figure 6). Treadmill walking with the Ekso GT™ elevated HR to a significantly greater level (100 ± 13 bpm, *p* = 0.005; ES = 0.5, medium) compared to treadmill walking without the Ekso GT™ (80 ± 20 bpm) at 1 km·h^−1^. A significant difference was present when comparing HR data when walking with the Ekso GT™ (112 ± 13 bpm, *p* = 0.002; ES = 0.7 medium) versus walking without the Ekso GT™ (83 ± 17 bpm) at 2 km·h^−1^. When walking at 3 km·h^−1^, HR was significantly higher during exoskeleton treadmill walking (133 ± 19 bpm, *p* = 0.002; ES = 0.8, large), in comparison to non-exoskeleton treadmill walking (88 ± 20 bpm). Walking with the Ekso GT™ resulted in a greater mean HR percentage increase (12%) from 1 to 2 km·h^−1^ than that of walking without the Ekso GT™ (3.8%). A higher mean HR percentage increase was also recorded between 2 and 3 km·h^−1^ when walking with the Ekso GT™ (18.8%) compared to walking without the Ekso GT™ (6%).

### 3.7. RPE

**Figure 7:**
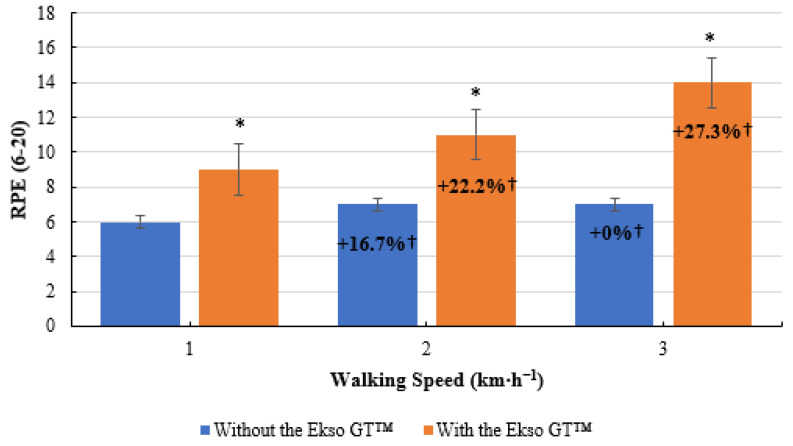
Rate of perceived exertion (RPE) scores during treadmill walking with and without Ekso GT™. * Significantly greater (*p* ≤ 0.05) when walking with Ekso GT™ versus walking with Ekso GT™. † Mean percentage RPE increase at each walking phase with and without Ekso GT™.

RPE scores were significantly greater during treadmill walking with the Ekso GT™ (9 ± 1, *p* = 0.011; ES = 0.8, large) compared to treadmill walking without the Ekso GT™ (6 ± 1) at 1 km·h^−1^. When walking at 2 km·h^−1^, RPE results were significantly higher during exoskeleton treadmill walking (11 ± 2, *p* = 0.011; ES = 0.8, large) in comparison to non-exoskeleton treadmill walking (7 ± 1). A significant difference was also present between RPE scores when walking with the Ekso GT™ (14 ± 1, *p* = 0.011; ES = 0.9, large) versus walking without the Ekso GT™ (7 ± 2) at 3 km·h^−1^. A greater mean RPE percentage increase was present between 1 km·h^−1^ and 2 km·h^−1^ when walking with the Ekso GT™ (22.2%) compared to walking without the Ekso GT™ (16.7%). A further increase of 27.3% was present between 2 km·h^−1^ and 3 km·h^−1^ when walking with the Ekso GT™; no increase in RPE scores was observed between 2 and 3 km·h^−1^ when walking without the Ekso GT™ (Figure 7).

## 4. Discussion

The aim of this research was to assess and compare the cardiorespiratory demands of treadmill walking with and without the use of the Ekso GT™. Results supported the proposed hypothesis that treadmill walking with the Ekso GT™ elevated the cardiorespiratory measurements ( $\dot{V}$ O_2_·kg^−1^, $\dot{V}$ CO_2_·kg^−1^, RER, $\dot{V}$ _E_, HR, and RPE) to a greater level than treadmill walking did without the Ekso GT™ among able-bodied participants. All cardiorespiratory findings demonstrated a medium-to-large ES except RER at 1 and 2 km·h^−1^ [32].

Cardiorespiratory trends reported in the current study support the findings of the existing literature [17,18,30]. $\dot{V}$ O_2_·kg^−1^ recorded during both walking bouts at 3 km·h^−1^ presented a similar trend to the findings reported by Chang et al. [17]; who highlighted that $\dot{V}$ O_2_·kg^−1^ during exoskeleton walking (22.5 ± 3.4 mL·kg^−1^·min^−1^) was significantly greater (*p* < 0.001) than that of non-exoskeleton walking (11.7 ± 2.0 mL·kg^−^^1^·min^−1^) while walking at a faster mean speed of 1.2 ± 0.2 m·s^−1^ (4.32 km·h^−1^) with, and 1.3 ± 0.2 m·s^−1^ (4.68 km·h^−1^) without the exoskeleton [17]. Treadmill walking with the Ekso GT™ at speed of 1 km·h^−1^ produced a greater mean $\dot{V}$ O_2_·kg^−1^ (10.9 ± 1.4 mL·kg^−1^·min^−1^) compared to overground walking with the Ekso GT™ (8.5 ± 0.90 mL·kg^−1^·min^−1^) at a slower mean speed of 13.8 ± 6.0 m·min^−1^ (0.83 km·h^−^^1^) within a SCI cohort [18]. Conversely, treadmill walking without the Ekso GT™ at 3 km·h^−1^ produced lower mean $\dot{V}$ O_2_·kg^−1^ (9.4 ± 1.4 mL·kg^−1^·min^−1^) results compared to an able-bodied control group during non-exoskeleton overground walking (11.3 ± 1.30 mL·kg^−1^·min^−1^) at a faster mean speed of 78.0 ± 10.5 m·min^−1^ (4.68 km·h^−1^) [18]. Asselin et al. [30] examined $\dot{V}$ O_2_·kg^−1^ during overground exoskeleton walking among a SCI population who walked at a mean speed of 0.22 ± 0.11 m·s^−1^ (0.79 km·h^−^^1^) using the ReWalk exoskeleton, which produced a mean $\dot{V}$ O_2_·kg^−1^ of 11.2 ± 1.7 mL·kg^−1^·min^−1^; which are similar to the current findings during treadmill walking with the Ekso GT™ at 1 km·h^−1^ (10.9 ± 1.4 mL·kg^−1^·min^−1^). Therefore, treadmill walking with the Ekso GT™ in an able-bodied population produced a similar $\dot{V}$ O_2_·kg^−1^ trend when compared to overground exoskeleton walking at various speeds conducted in previous research [17,18,30].

Similarly, treadmill walking with the Ekso GT™ elevated $\dot{V}$ CO_2_·kg^−1^ and $\dot{V}$ _E_ to a significantly greater level (*p* ≤ 0.05) in comparison to treadmill walking without the Ekso GT™ at the same fixed speeds (1, 2, and 3 km·h^−1^). RER recordings during treadmill walking with the Ekso GT™ supported the findings of existing research, which demonstrated that carbohydrate utilisation was greater during exoskeleton walking versus non-exoskeleton walking [14,33,34]. Fat was the main fuel source when walking without the Ekso GT™ as RER was closer to 0.7 (0.80 ± 0.10 to 0.80 ± 0.07) [35]. Carbohydrate was the primary fuel source for energy metabolism when walking with the Ekso GT™ as RER was closer to 1.0 (0.84 ± 0.12 to 0.96 ± 0.15) [35]. Thus, an elevation in exercise intensity was observed during exoskeleton treadmill walking versus non-exoskeleton treadmill walking at the same fixed speeds, as fat oxidation decreases, and carbohydrate utilisation increases with an increase in exercise intensity [36]. Therefore, when using the Ekso GT™, the glycolytic energy system was the primary pathway used for energy metabolism [37]; this may promote several favourable health and cardiorespiratory fitness adaptations [38,39,40,41,42,43,44,45]. RPE scores reported in the current study indicated that participants reached at least a moderate-intensity level of aerobic activity during treadmill walking with the Ekso GT™ at 3 km·h^−1^ (14 ± 1, ‘somewhat hard’ to ‘hard’), which was significantly greater (*p* = 0.011) than the RPE scores when walking without the Ekso GT™ at the same speed (7 ± 2, ‘very, very light’). The current RPE findings presented a similar trend when compared to existing literature, which demonstrated that participants rated exoskeleton walking as “moderate to somewhat hard” [14,30,46]. In contrast, Kressler et al. [33] highlighted that exoskeleton gait training can only produce light exercise intensity. However, exercise intensity was recorded throughout a one-hour session in which participants rested as required to avoid fatigue; therefore, the implemented methodology may be responsible for the discrepancy in exercise intensity [33].

HR data collected during treadmill walking with the Ekso GT™ at 1 km·h^−1^ (100 ± 13 bpm), 2 km·h^−1^ (112 ± 13 bpm) and 3 km·h^−1^ (133 ± 19 bpm) in the current study aligned with existing research conducted within a SCI cohort during overground exoskeleton walking (118 ± 21 bpm) at 0.79 km·h^−1^ [30]. Similarly, Chang et al. [17] highlighted that exoskeleton walking at 4.32 km·h^−1^ (148 ± 19 bpm) produced a considerably higher HR than non-exoskeleton overground walking (99 ± 14 bpm) did at 4.68 km·h^−1^. Chang et al. [17] reported higher HR results than those of the current study during exoskeleton walking. However, this may be attributed to the 1.68 km·h^−1^ difference in walking speed or differences in the exoskeleton devices. Previous research also demonstrated increased HR data during powered exoskeleton assisted walking, and produced similar results (100 ± 13, 88–134, 72–132, and 115 bpm within a single participant) when compared to the current findings [11,14,15,47,48]. Evans et al. [16] recorded HR over two exoskeleton walking bouts within a SCI population. Mean walking speed during the first walking bout was 0.19 ± 0.01 m·s^−1^ (0.68 km·h^−1^), which produced a mean HR of 121 ± 30 bpm; and mean walking speed during the second walking bout was 0.27 ± 0.05 m·s^−1^ (0.97 km·h^−1^) produced a mean HR of 142 ± 35 bpm [16]. Kwon et al. [49] collected HR data during 6 MWT (112.5 ± 13.6 bpm) and 30 MWT (118.6 ± 14.6 bpm) using the ReWalk exoskeleton within a SCI cohort, walking at mean speeds of 6.0 ± 2.4 m·min^−1^ (0.36 km·h^−^^1^) and 6.6 ± 1.2 m·min^−1^ (0.4 km·h^−1^) respectively [49]. When compared to the current study, higher HR results were reported by Evans et al. [16] during the second walking bout and by Kwon et al. [49] whilst walking at slower speeds of 0.97 km·h^−1^ and 0.4 km·h^−1^ respectively, this may be due to the differences in devices used, the demands of overground walking versus treadmill walking, the difference in population, or differences in cardiorespiratory fitness levels. The most common formula used to predict maximal HR (MHR) is: 220—age (years) [50]. Using this formula, Chang et al. [17] reported that participants reached 85.3 ± 12.6% of MHR; when applying the same formula, one participant in the current study reached 94.5% (189 bpm) of MHR during treadmill walking with the Ekso GT™ at 3 km·h^−1^. However, this formula often overestimates MHR when compared to true MHR [51], which is most commonly assessed using a maximal graded treadmill assessment [52]. The 220—age (years) formula was originally proposed for individuals ≥60 years old with chronic pathologies and at a high risk of developing cardiorespiratory disease [53]; therefore, may not be applicable to a wider population.

High-intensity exercise is safe to employ within special populations; it may elicit more favourable cardiorespiratory fitness adaptations, and improve blood pressure and insulin sensitivity more effectively than lower-intensity exercise [54,55]. High-intensity exercise may contribute to lipolysis and subsequently reduce body fat mass [56]. Reducing body fat mass may mitigate the likelihood of developing cardiorespiratory disease and diabetes, as adipose tissue is a precursor to these conditions [57]. Central (increased stroke volume) and peripheral adaptations (increased capillary density and mitochondrial adaptation) may be responsible for the increase in cardiorespiratory fitness elicited by high-intensity exercise [58,59,60]. Therefore, once medically cleared to undertake regular PA, exoskeleton walking may enable individuals with neurological impairments to exercise at a high intensity, and promote more favourable cardiorespiratory fitness and body composition adaptations than an arm crank or wheelchair ergometer can, as exoskeleton walking may elevate thoracohumeral and trunk muscle mass activation rather than isolating the upper extremities [14].

Additionally, prior to walking with and without the Ekso GT™, RBP and RHR were recorded. Mean RBP and RHR returned to or below baseline scores prior to walking with the Ekso GT™; therefore, participants were fully rested and recovered following walking without the Ekso GT™, as systolic BP and HR normally return to resting levels within a few minutes postexercise [61]. Therefore, results gathered when walking with the Ekso GT™ were not predetermined by any fatigue accumulated when walking without the Ekso GT™. The current study demonstrated how exoskeleton training enabled able-bodied participants to significantly increase cardiorespiratory parameters and adds to the emerging existing body of literature which suggests that powered exoskeletons may be an appropriate support method to help elevate cardiorespiratory measurements and may provide adequate stimulus to elicit favourable cardiorespiratory fitness adaptations [16]. According to Chang et al. [17], exoskeleton walking at a similar speed within a neurologically compromised cohort would likely further elevate the cardiorespiratory response. As individuals with neurological impairments have limited lower limb function, a greater demand could potentially be placed on the upper extremities to maintain balance and stability, thus increasing cardiorespiratory response [17]. Participants in the current study had full lower-extremity support to maintain balance, and less effort was required from the upper extremities. 

### 4.1. Practical Implications

The current findings may be transferrable to individuals with neurological impairments. Regular use of the Ekso GT™ throughout a rehabilitation programme may enable individuals to increase PA levels, while aiming to meet WHO PA guidelines for health [12]. The use of the Ekso GT™ may also promote further health benefits and enhance QoL [62], by improving strength, cardiorespiratory fitness, range of motion, mobility, and overall physical and mental health while reducing the risk of developing cardiorespiratory related diseases and their comorbidities [7,12,63,64,65]. Furthermore, improving QoL may lead to reduced stress levels and enhanced mood, self-satisfaction, and self-esteem [66,67], increased self-confidence self-image and sleep quality [66,68], improved psychological and emotional state [69], and increased participation in social activities [66].

### 4.2. Limitations

Although the current sample size (*n* = 8) is limited, the number of included participants met the requirements of the power calculation (*n* = 7; study power: 0.98); therefore, this study obtained an adequate number of participants to allow for the findings to be declared significant [70]. ES was calculated to determine the meaningfulness of the data. Several cardiorespiratory parameters presented medium to large effect sizes, and therefore results may be generalisable as they were not directly impacted by sample size [70]. Despite the involved population throughout this study being able-bodied, the findings may be plausible to individuals with neurological impairments [70], as the current findings aligned with the results reported within SCI populations in which exoskeleton-assisted walking also resulted in an elevation of cardiorespiratory parameters [14,16,18,30]. Treadmill walking may limit how the results can be interpreted, as it is often argued that treadmill walking at a pre-set speed does not accurately replicate the demands of overground walking [71]. However, the current findings during exoskeleton treadmill walking were supported by the results of the existing literature during overground exoskeleton walking [17].

Future research should first use larger samples to confirm the results of the current study and the existing literature consisting of smaller samples. Second, individuals with neurological impairments should be included to support the current findings while also using a control group as a reference point. Third, cardiorespiratory demands of overground walking with the Ekso GT™ should be examined, as pre-set treadmill-walking speed may not accurately replicate overground walking [71], as the treadmill may artificially improve gait rhythm, and users may not reach overground walking speed [72]. Considerations for further research should include the longitudinal effects of exoskeleton-based rehabilitation on cardiorespiratory function, gait function, and secondary health conditions with the primary aim of improving health and overall QoL.

## 5. Conclusions

In conclusion, one group of healthy able-bodied participants (*n* = 8) completed two 12 min treadmill walking bouts, one with and one without the Ekso GT™, at the same fixed speed. Cardiorespiratory parameters were recorded throughout both walking bouts. Treadmill walking with the Ekso GT™ elevated all measurements to a significantly greater level when compared to treadmill walking without the Ekso GT™ (except RER at 1 km·h^−1^; *p =* 0.230) within an able-bodied population. The increase in cardiorespiratory parameters may provide adequate stimulus to the cardiorespiratory systems to cause favourable adaptations in response to exercise. In turn, this may increase cardiorespiratory fitness, enhance walking ability, increase muscular strength, and improve health [13,21,73,74]. Walking with the Ekso GT™ also enabled participants to reach at least a moderate-intensity level of exercise; therefore, prolonged bouts of exoskeleton walking may be an appropriate method to enable neurologically compromised individuals to achieve the PA guidelines for health, which may subsequently elicit additional health benefits [12]. Therefore, incorporating a powered exoskeleton into a rehabilitation programme may strengthen rehabilitation practices by providing appropriate stimulus to enhance cardiorespiratory fitness.

## Figures and Tables

**Table 1:** Inclusion and exclusion criteria.

Inclusion	Exclusion
≥18 and ≤70 years old	Any current contractures, fractures, or joint dislocations in the lower limbs.
Neutral ankle dorsiflexion	Osteoporosis
Passive range of motion (0–120° knee flexion; 0–90° hip flexion; 0–10° hip extension)	Body mass > 100 kg
Height ≥ 157.5 cm ≤ 188 cm	Unequal leg length
No orthopaedic or neurosurgery within the last six months	Unhealed skin lesions in the lower limbs
Stable cardiorespiratory system	Thromboembolic or cardiorespiratory diseases

## Data Availability

The data presented in this study are available on request from the corresponding author.
